# microRNA expression in the prefrontal cortex of individuals with schizophrenia and schizoaffective disorder

**DOI:** 10.1186/gb-2007-8-2-r27

**Published:** 2007-02-27

**Authors:** Diana O Perkins, Clark D Jeffries, L Fredrik Jarskog, J Michael Thomson, Keith Woods, Martin A Newman, Joel S Parker, Jianping Jin, Scott M Hammond

**Affiliations:** 1 Department of Psychiatry, University of North Carolina at Chapel Hill, CB 7160, Chapel Hill, NC 27599, USA; 2 School of Pharmacy, University of North Carolina at Chapel Hill, CB 7360, Chapel Hill, NC 27599, USA; 3 Renaissance Computing Institute, University of North Carolina at Chapel Hill, Chapel Hill, NC 27599, USA; 4 Department of Cell and Developmental Biology, University of North Carolina at Chapel Hill, CB 7090, Chapel Hill, NC 27599, USA; 5 Constella Group, LLC, Meridian Parkway, Durham, NC 27713, USA; 6 Department of Molecular Biology, University of North Carolina at Chapel Hill, CB 7104, Chapel Hill, NC 27599, USA

## Abstract

**Background:**

microRNAs (miRNAs) are small, noncoding RNA molecules that are now thought to regulate the expression of many mRNAs. They have been implicated in the etiology of a variety of complex diseases, including Tourette's syndrome, Fragile × syndrome, and several types of cancer.

**Results:**

We hypothesized that schizophrenia might be associated with altered miRNA profiles. To investigate this possibility we compared the expression of 264 human miRNAs from postmortem prefrontal cortex tissue of individuals with schizophrenia (*n *= 13) or schizoaffective disorder (*n *= 2) to tissue of 21 psychiatrically unaffected individuals using a custom miRNA microarray. Allowing a 5% false discovery rate, we found that 16 miRNAs were differentially expressed in prefrontal cortex of patient subjects, with 15 expressed at lower levels (fold change 0.63 to 0.89) and 1 at a higher level (fold change 1.77) than in the psychiatrically unaffected comparison subjects. The expression levels of 12 selected miRNAs were also determined by quantitative RT-PCR in our lab. For the eight miRNAs distinguished by being expressed at lower microarray levels in schizophrenia samples versus comparison samples, seven were also expressed at lower levels with quantitative RT-PCR.

**Conclusion:**

This study is the first to find altered miRNA profiles in postmortem prefrontal cortex from schizophrenia patients.

## Abstract 2

Transcriptional profiling reveals a possible association between schizophrenia and altered miRNA expression

## Background

Schizophrenia is a common neuropsychiatric disorder affecting one percent of the general population. The personal, familial, and societal costs of the disease are enormous, with chronic symptoms that result in marked functional disability. In fact, approximately three percent of all person-years lived with disability are due to schizophrenia [1].

It is clear that schizophrenia has a strong genetic component, although its genetic basis remains unknown [2]. Consistent with a disease mechanism that involves post-transcriptional dysregulation of gene expression, postmortem studies find altered levels of mRNA and proteins rather than a specific abnormal protein [3]. Postmortem studies also find differences between schizophrenia and unaffected comparison subjects in the relationship of such mRNAs and cognate proteins [4,5].

microRNAs (miRNAs) are a class of noncoding RNAs (ncRNAs) that in animals regulate gene expression by inhibiting mRNA translation. Each miRNA is initially processed from a large (approximately 200 nucleotide (nt) to several thousand nt) RNA transcript, the 'primary miRNA' (pri-miRNA) to a smaller (approximately 58-137 nt) hairpin precursor miRNA (pre-miRNA) by a protein complex, the 'microprocessor', and then by *DICER1 *(alias Dicer) to the mature miRNA [6]. The mature miRNA joins with the RNA-induced silencing complex (RISC), and then binds the RISC to a partially complementary target region in an mRNA to accelerate mRNA degradation or inhibit translation. Some 474 RNA hairpins (pre-miRNAs) are known to be transcribed in humans, yielding 471 distinct, mature miRNAs, and there are in addition over 800 predicted human miRNAs. The associated control systems might regulate expression of thousands of human genes [7-9]. In particular, seminal experiments have shown that miRNAs regulate a variety of key biological functions, including cell proliferation and differentiation [10-15], insulin secretion [16], and apoptosis [17]. Emerging evidence suggests that miRNAs also regulate brain development [18,19], dendritic spine morphology [20], and neurite outgrowth [21], that is, certain processes that are hypothesized to be associated with schizophrenia neuropathology.

In addition to critical regulatory roles in development and cellular functions, miRNAs have now been implicated in several human diseases [22]. For example, the etiology of some cases of Tourette's syndrome, a disorder characterized by vocal and motor tics, has been shown to be related to either the absence of or a mutation in the miR-189 target site in the 3' untranslated region (UTR) of gene *SLITRK1 *[23]. Fragile X syndrome, one of the most common genetic disorders affecting brain function, is characterized by deficits that range from learning disabilities in individuals with normal intelligence to severe intellectual deficits and behavioral disturbances. The genetic basis is most commonly a CGG repeat expansion in the 5' UTR of *FMRP *causing transcriptional silencing [24]. *FMRP *might regulate the translation of mRNAs through association with RISCs and miRNAs, and, in particular, might regulate translation of mRNAs locally in the dendrites [24-26].

Given the critical role that miRNAs might play in regulating brain development early in life and mediating synaptic plasticity later in life, we have hypothesized that the etiopathology of schizophrenia might be associated with altered expression or function of miRNAs [27]; the association might be causative or part of compensatory reactions to some other causative agents. As a first step we compared the expression of human miRNAs from postmortem prefrontal cortex (PFC) of individuals with schizophrenia to that of unaffected individuals.

## Results

### General description of prefrontal cortical miRNA expression

From the 265 distinct, human miRNAs included on our array, 244 were detected (1.5-fold over background) in the PFC tissue of ≥60% of the study subjects. These included robust detection of miRNAs previously known to be expressed in the brain (for example, let-7a to let-7i) as well as brain-specific miRNAs (for example, miR-124a and miR-125b) (Additional data file 1) [11,28].

### miRNA expression in schizophrenia versus unaffected comparison subjects

**Figure 1:**
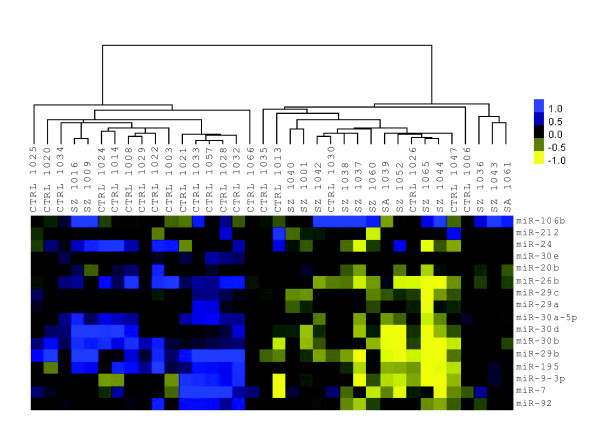
An miRNA expression map shows differentiated genes as determined by SAM analysis. Yellow indicates low expression and blue indicates high expression, relative to the median.

Assuming a false discovery rate (FDR) of 5%, 16 miRNAs were differentially expressed in PFC of schizophrenia subjects (*n *= 13) or schizoaffective disorder (*n *= 2) versus PFC of 21 psychiatrically unaffected individuals (Table 1). Of the 16 distinguished miRNAs, 15 were expressed at lower (fold change 0.63 to 0.89) and one at higher (fold change 1.77) levels than in the psychiatrically unaffected comparison subjects. A heat map based on cluster analysis illustrates the differentiated expression levels of these probes (Figure 1). Controlling on brain pH, postmortem interval (PMI), and hemisphere, and excluding the two subjects with schizoaffective disorder from the analyses did not substantially affect these results (Additional data file 2).

### Quantitative RT-PCR verification of microarray results

**Figure 2:**
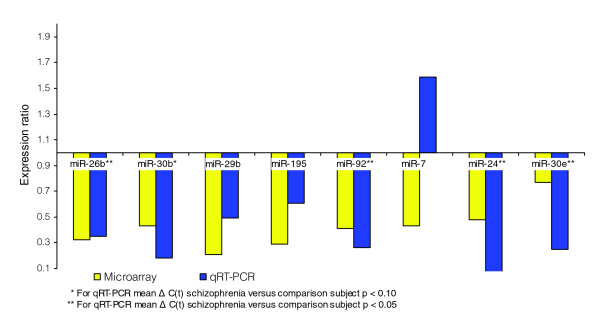
miRNA microarray fold changes can be compared with delta-delta C(t) functions of qRT-PCR data (see Materials and methods). The comparisons are over four samples from schizophrenia patients and four samples from psychiatrically unaffected comparison subjects. Seven of the eight comparisons are consistent.

The expression levels of 12 selected miRNAs were also determined by quantitative RT-PCR (qRT-PCR) in our lab (Additional data file 3). For the eight miRNAs distinguished by being expressed at lower microarray levels in schizophrenia samples versus comparison samples, seven were also expressed at lower levels with qRT-PCR (Figure 2). For four of the seven, the difference in expression was significant with *p *< 0.05, consistent with microarray findings for the same miRNAs. The eighth miRNA, miR-7, was found by qRT-PCR to have higher levels in schizophrenia than comparison subjects, but the difference in expression was not significantly different between groups (*p *= 0.23); we have not determined a cause for this one discrepancy of PCR versus microarray results. We also compared expression of four miRNAs that were not differentially expressed in the microarray results, and found none to be differentially expressed by qRT-PCR (*p *> 0.05).

### Effect of haloperidol exposure on miRNA expression

Since all of the schizophrenia subjects were treated or had previously been treated with antipsychotics and none of the psychiatrically unaffected subjects were reported to have such a treatment history, we endeavored to evaluate the effect of antipsychotic treatment on miRNA expression. We compared expression of 179 rat miRNAs in haloperidol-treated and -untreated rats. With a FDR of 5% we found that three miRNAs were expressed at higher levels in the haloperidol-treated rats: miR-199a, miR-128a, and miR-128b. None of these miRNAs was differentially expressed in the PFC of schizophrenia patients (Additional data file 4).

### miRNA and Affymetrix U133A probe relationships

We considered whether the observed pattern of lower expression of some miRNAs in schizophrenia subjects was related to lower pri-miRNA transcription. We adopted the previously published method of Thomson and colleagues [29], where the pri-miRNA expression was determined from existing archived mRNA microarray results from the PFC of the same study subjects. A total of 52 of the miRNAs included in this study could be mapped to a primary transcript that was present among the mRNA transcripts accessible with the Affymetrix U133A array. All but three of the miRNAs with corresponding U133A probes were from the introns of protein-coding genes (host genes). The mean expression of only two of the Affymetrix U133A probes was significantly different between groups (*ELM2 *hosting miR-330 with *p *= 0.03; *MYH6 *hosting miR-208 with *p *= 0.03). However, these differences were not significant after correction for multiple comparisons (*p *> 0.05).

We then focused on the five miRNAs expressed at significantly lower levels in schizophrenia that also had a U133A probe that included the pri-miRNA transcript (miR-26b, miR-9-3p (alias miR-9*), miR-24, miR-7, and miR-30e). The ratio of mature miRNA to primary miRNA transcripts was lower for schizophrenia versus controls for all 5 miRNAs, and the difference in ratios reached statistical significance for 3 of the 5 (miR-26b, *p *= 0.009; miR-9-3p, *p *= 0.002; and miR-24, *p *= 0.037). For the one miRNA that was expressed at a significantly higher level in schizophrenia subjects, miR-106b, the ratio was also significantly higher (*p *= 0.003 and *p *= 0.006 for the two associated Affymetrix pri-miRNA probes). In the remaining 46 miRNAs with a corresponding Affymetrix U133A probe for their pri-miRNA transcripts, the ratio of miRNA to host mRNA was significantly lower for two pri-miRNAs (primary transcripts for miR-218, *p *= 0.021, and miR-9, *p *= 0.006) and significantly higher for five (miR-482, *p *= 0.015; miR-190, *p *= 0.018; miR-105, *p *= 0.02; miR-148b, *p *= 0.027; miR-218, *p *= 0.02). Thus, we found that the miRNA:U133A probe ratios of the schizophrenia group were significantly different from those of the comparison group for 4 of the 6 differentiated miRNAs but only 7 of the 46 nondifferentiated miRNAs (*p *= 0.013, Fisher's exact test) (Additional data file 5).

### Common motifs near the pre-miRNA:pri-miRNA junction

We hypothesized that the system regulating processing of the pri-miRNA to pre-miRNA might involve a motif within the pri-miRNA and upstream of the single-stranded RNA (ssRNA)-double-stranded RNA (dsRNA) junction that would lend selectivity to this process. Specifically, we hypothesized that an upstream motif of some kind is shared by the 15 miRNAs that were found to be downregulated in our tests of schizophrenia PFC samples. To seek bioinformatic indications, we focused on source pre-miRNAs that were isolated (no other pre-miRNAs within 1,000 bases), yielding 11 distinguished, isolated pre-miRNAs: miR-7-1, miR-7-2, miR-7-3, miR-9-1, miR-9-2, miR-9-3, miR-26b, miR-30a, miR-30b, miR-30d, miR-30e. Of these, miR-9-1 and miR-30a can yield two mature miRNAs; the others yield one. Furthermore, miR-7-2, miR-9-2, miR-9-3, miR-30b, and miR-30d are intergenic, and the others are intronic in coding genes.

**Figure 3:**
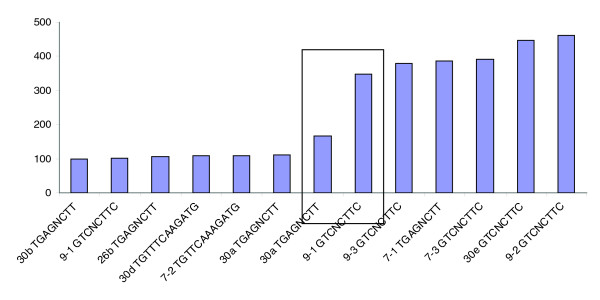
Regarding the 11 isolated miRNAs distinguished in schizophrenia, this figure shows the distances (numbers of bases) from shared 5' motifs we discovered (two 8 nt and two 12 nt motif sequences in the pri-miRNA) to the ssRNA-dsRNA junctions at starts of pre-miRNAs. Pre-miR-30a and -9-1 have double motif instances; second instances are in the rectangle. Ignoring the second instances as redundant leaves some motif distances in sharp clusters.

Using a combination of approaches, we found the motif UGAGNCUU upstream of pre-miRNA sequences for miR-26b, miR-30a, miR-30b, and miR-7-1. We also found GUCNCUUC upstream of pre-miRNAs miR-9-1, miR-9-2, miR-9-3, miR-7-3, and miR-30e. Thus, both 8 nt motifs are found upstream of 9 of the 11 isolated, distinguished pre-miRNAs. Lastly, instances of UGUUNNAAGAUG were found upstream of pre-miRNAs for miR-30d and miR-7-2 at the same distance, 108 bases, and not within 500 bases upstream of any other human, isolated pre-miRNAs. For displays of the motifs and the bases between motifs and junctions, see Additional data file 6; clustering of the number of bases in each such interval is displayed in Figure 3. Bioinformatic searches by us have found neither shared motifs that are positioned at similarly clustered distances from the junctions nor strong general homology among the 11 upstream regions.

Importantly, the same 8 nt motifs UGAGNCUU and GUCNCUUC are absent from the 500-base 5' regions of most undistinguished pre-miRNAs. That is, the same motifs are also upstream of only 13 isolated, undistinguished pre-miRNAs among a total of 192 isolated, human pre-miRNAs, and some of the 13 are sequentially similar as mature miRNAs to the 11 distinguished ones. However, carefully designed and executed *in vivo *experiments would be needed to determine whether the above or any other motifs are actually functional; the above motifs are intriguing, but their bioinformatic properties are certainly not a proof of common regulation of coordinated pre-miRNA excision.

## Discussion

miRNAs, with their key roles in regulating both synaptic plasticity and brain development, are candidate genetic contributors to the etiopathology of schizophrenia. miRNA expression for 16 miRNAs was significantly different in the PFC of schizophrenia versus comparison subjects, with all but one of the differentiated miRNAs decreased in the schizophrenia subjects. To our knowledge this study is the first to associate altered expression of miRNAs with schizophrenia. Possibly the association is etiologic, but it could also be part of a complex response to other factors.

### A hypothesized role for altered miRNA biogenesis

**Figure 4:**
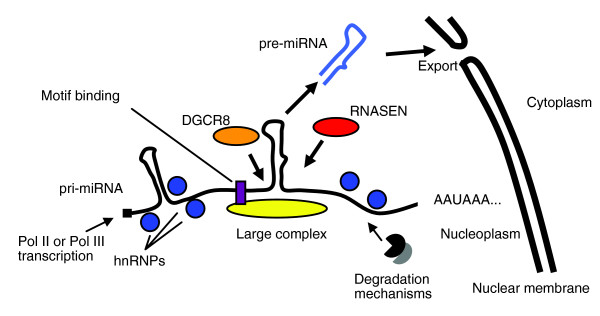
Transcription yields a continuous supply of some types of pri-miRNA transcripts, capped and polyadenylated. hnRNPs are hypothesized to shape the pri-miRNA into linear and hairpin sections. A signaling system somehow recruits and activates unknown factors that select particular pre-miRNA hairpins on a particular pri-miRNA for excision and processing in the miRNA pathway. We hypothesize that this system might include a binding motif. RNASEN and DGCR8 are products of genes 29102 and 54487.

Our follow-up analyses were designed to generate hypotheses about possible mechanisms that could explain the downregulation of miRNAs reported in this study. For miRNAs hosted in introns of coding genes, we found that the ratios of microarray expression levels of miRNA versus mRNA (of host gene) were significantly different for miRNA distinguished by schizophrenia. That is, 4 of the 6 hosted, distinguished miRNAs exhibited the difference, but only 7 of the 46 undistinguished miRNAs did so. This suggests a role for altered biogenesis of miRNAs rather than altered transcription of pri-miRNAs. In addition, our bioinformatic investigations found 2 common motifs located at approximately 100 or approximately 400 bases from the pri-miRNA:pre-miRNA junction in 9 of the 11 isolated, distinguished miRNAs; but the same motifs are absent in almost all of the undistinguished miRNAs. We speculate (see Figure 4) that these motifs might represent binding sites for factors like heteronuclear ribonuclear proteins (hnRNPs) [30], known to chaperone other RNA events.

The bioinformatic similarities involving motifs, though not yet investigated *in vivo*, are consistent with the hypothesis that the coordinated downregulation of 15 miRNAs reported in this study might be related to alternative processing during the pre-miRNA biogenesis process, rather than altered pri-miRNA transcription. There is evidence that, in some cases, miRNA biogenesis regulates mature miRNA levels. Thomson *et al*. [29] found that in mice, levels of mature miRNAs hsa-let-7g and hsa-let-7f-2/miR-98 increased over 4,000-fold in day 14.5 embryos from levels in embryonic stem cells. However, over the same developmental period the primary transcript pri-miRNA expression levels did not change, and pre-miRNA levels were essentially undetectable. Also, the same Thomson analysis indicates that the widespread downregulation of miRNAs observed in cancer [31,32] might be due to a failure in miRNA processing that is post-transcriptional (transcription of pri-miRNA). Discovery of parallel mechanisms of regulation of other sets of miRNAs, such as the 15 downregulated miRNAs in schizophrenia, would, therefore, be of considerable interest.

Further study is required to test the hypothesis that altered regulation of miRNA biogenesis might be involved in the etiopathology of schizophrenia, and whether the above motifs are involved in regulating miRNA processing from pri-miRNA to pre-miRNA.

As a final note, DiGeorge critical region 8 (*DGCR8*), involved in miRNA biogenesis as a component of the microprocessor, is located in a genomic region of chromosome 22q11 where microdeletions have been associated with a 30-fold increased risk of schizophrenia [33,34]. Microdeletions in 22q11 occur in approximately 1 in 3,000 live births but are present in 0.5% to 3% of individuals with schizophrenia [35,36]. Possibly, *DGCR8 *polymorphisms that alter expression or function through haploinsufficiency or other genetic variants might also contribute to the etiopathology of schizophrenia by impacting miRNA biogenesis and regulation of gene expression.

### Common potential mRNA targets

Dysregulation of miRNA levels would be anticipated to affect the translation of multiple protein coding genes. Bioinformatic strategies are now developed to identify potential miRNA target sites in the 3' UTR of a protein coding gene, for example the program miRanda [7]. The potential targets of miRNAs often include hundreds of genes because the reverse complement of some 'seeds' (bases 2 through 8 of the mature miRNA) appears in multiple locations in many pre-mRNA 3' UTRs. However, only a few of these potential target sites have been verified as potent *in vivo *[37]. With the understanding that identification of mRNA targets is speculative, we explored whether there might be common mRNA targets for the 15 distinguished, downregulated miRNAs and whether these targeted genes are over-represented in any Kyoto Encyclopedia of Genes and Genomes (KEGG) pathway through the KEGG website [38].

The differentially expressed miRNAs are currently annotated in the Memorial Sloan-Kettering Cancer Center Computational Biology Center web site. These 15 miRNAs are identified using miRanda to potentially target the 3' UTRs of over 4,600 genes, with 1,539 targeted by 2 or more of them [39]. Using the programs offered by the Database for Annotation, Visualization, and Integrated Discovery (DAVID) to identify over-represented pathways, we found that the genes that were commonly targeted by the miRNAs were significantly clustered in 12 KEGG pathways (Table 2) [40]. It is of interest that the most significantly differentiated pathways are involved in synaptic plasticity at the level of dendritic spines. For example, the *MAPK *and phosphatidylinositol signaling pathways are involved in the regulation of dendritic spine morphogenesis, size, and shape [41,42] and act through regulation of the actin cytoskeleton [43]. In addition, the focal adhesion pathways mediated through extracellular matrix receptor interactions have also been shown to control dendritic spine plasticity [44]. Translation of mRNA into proteins that are important to synaptic plasticity can occur locally in dendrites [45]. Thus, the miRNAs differentiated in this study might be involved in the regulation of synaptic plasticity, and in that manner associated with characteristics of synaptic plasticity in schizophrenia.

## Conclusion

Although the functions of most human miRNAs have yet to be discovered, miRNAs have emerged as key regulators of gene expression. The findings of this study implicate a role for miRNAs in schizophrenia, and lead us to the hypothesis that there is altered processing of miRNAs during the miRNA biogenesis process in schizophrenia. This hypothesis is analogous to that for altered miRNA transcription in cancer by Thomson *et al*. [29].

## Materials and methods

### Postmortem tissue

This study was approved by the Institutional Review Board of the University of North Carolina School of Medicine. Postmortem human brain tissue was obtained from the Harvard Brain Tissue Resource Center [46]. Tissue consisted of frozen blocks (300-500 mg/block) from the PFC (Brodmann area nine from 15 individuals with schizophrenia and 21 unaffected comparison subjects (Table 3)). The tissue was group-matched for age, gender, PMI, and hemisphere. Postmortem neuropathological examinations were performed by an experienced neuropathologist, and all subjects included in the collection were free of neurodegenerative pathology. Postmortem neurotoxicological studies showed no evidence of illicit substance use at the time of death.

### Animals

Experimental protocols were approved by the UNC Institutional Animal Care and Use Committee. Singly housed, male Sprague-Dawley rats (150-200 g; Charles River, Raleigh, NC, USA) received daily intraperitoneal injections of haloperidol 1 mg/kg/d (*n *= 6) or saline 0.9% (*n *= 6) for 4 weeks. One hour after the final dose, rats were briefly anesthetized with ether and sacrificed; their brains were removed and hemisected. Right anterior medial frontal cortex was dissected out and frozen on dry ice. All tissue was kept frozen at -80°C until use.

### miRNA microarray procedures

miRNA microarray expression analysis was performed as previously described [47]. Tissue disruption by Dounce homogenization was followed by total RNA isolation with TRIZOL™ reagent (Invitrogen, Carlsbad, California, USA). RNA (5 μg) was labeled with T4-RNA ligase and precipitated with 0.3 M sodium acetate, 2 volumes ethanol, and re-suspended in water.

Oligonucleotide probes were synthesized in duplicate for 264 human miRNAs antisense to the mature sequence reported in the Sanger miRNA registry [48]. Probes were spotted in duplicate on Corning (Corning, New York, USA) GAPS-2 coated slides using a robotic spotter and cross-linked by UV. Hybridization and washing were performed as described. All arrays were from the same batch, and the microarrays were run on the same day by the same two persons. Our prior research indicates that our in-house miRNA microarrays have excellent reliability and validity [49].

Microarray data analysis began with data extraction from the GPR files. Data points were eliminated if foreground was not 1.5 times local background and a probe was removed if >40% of the data points were missing. A total of 239 miRNA remained after this pre-processing. Data were background subtracted, log-transformed, and missing values were imputed using *k*-NN [50]. For comparisons across samples, data were normalized using rank invariant normalization [51]. The per-sample mean of the two rank invariant normalized probes was used for analyses. Univariate calculations of differential expression were estimated using Statistical Analysis of Microarrays (SAM; two-class, unpaired test; 500 permutations; FDR of 5%) [52]. All analysis procedures were done using R [53]. Cluster analysis was done with GeneCluster^© ^[54] and displayed using TreeView^© ^[55] (Figure 1).

### mRNA microarray analysis procedures

Previous to our research, mRNA microarray profiling of PFC tissue from these same subjects (but different samples) was performed at the Harvard Brain Tissue Resource Center with Affymetrix U133A^© ^arrays using standard methods and quality control procedures. The cel files and information on sample acquisition, preparation, and microarray analysis are publicly available and were downloaded from the Center's National Brain Databank.

The U133A microarrays were normalized using GC Robust Multi-Array (GCRMA), and analysis of probe expression levels was done with SAM. We used the March 2006 version of the UCSC Human (*Homo sapiens*) Genome Browser [56] to determine the U133A probes that corresponded to miRNA locations in host genes.

### qRT-PCR procedures

Total RNA (5 μg) was DNase I (Promega, Madison, Wisconsin, USA) treated according to the manufacturer's instructions, phenol:chloroform extracted, ethanol precipitated, and dissolved in DEPC-treated dH_2_O (DEPC; diethylpyrocarbonate). RNA (5 μg) was polyadenylated using Poly(A) polymerase (Ambion, Austin, Texas, USA) according to the manufacturer's instructions, phenol:chloroform extracted, ethanol-precipitated, and dissolved in DEPC-treated dH_2_O. A modified cDNA was made as follows: 5 μg of polyadenylated RNA was reverse-transcribed using Superscript II reverse transcriptase (Invitrogen, Carlsbad, California, USA) with 2.5 μg of random hexamers and 500 ng of oligo(dT) adapter primer (5'-GCGAGCACAGAATTAATACGACTCACTATAGGTTTTTTTTTTTTVN-3') according to the manufacturer's instructions. The reaction was terminated by incubation at 70°C for 10 minutes and diluted into 2 ml of dH_2_O (5 ng/μl). Quantitative PCR was used to measure the mature miRNA transcript as follows: 5 μl of cDNA was mixed with 5 pmol of both the forward and reverse primers in a final volume of 12.5 μl and mixed with 12.5 μl of 2× SYBR Green PCR master mix (Applied Biosystems, Foster City, California, USA). Primer sequences are in Additional data file 7. All reactions were run in triplicate on a DNA Engine Opticon 2 (Bio-Rad Laboratories, Hercules, California, USA). The amplification protocol for mature miRNA PCR was performed according to the high-stringency protocol of Shi and Chiang [57] except the reverse primer Mir-qPCR-3-3' (5'-GCAGCA CAGAATTAATACGACTCAC-3') was used in conjunction with an exact sequence-specific primer to each miRNA. Mature miRNA expression used the reference gene U6 snRNA (U6-F, 5'-CGCTTC GGCAGCACATATAC-3'; U6-R, 5'-TTCACGAATTTGCGTGTCAT-3'). The expression was determined for eight subjects, four with schizophrenia and four healthy subjects (Additional data file 3). Expression was calculated using the delta-delta C(t) method: 2^ΔCT healthy-ΔCT schizophrenia ^with ΔC_T _= (C_T _miRNA - C_T _reference RNA U6) [58].

## Additional data files

The following additional data are available with the online version of this paper. Additional data file 1 is a table listing the tested miRNAs and their expression levels and fold changes. Additional data file 2 is a table showing data conditioning on PMI, pH, and hemisphere. Additional data file 3 is a table of qRT-PCR results. Additional data file 4 includes characterization and microarray results on rats treated with haloperidol. Additional data file 5 is a table of host mRNA data. Additional data file 6 lists putative motifs within regions upstream of some distinguished pre-miRNAs. Additional data file 7 is a table listing primer sequences.

## Supplementary Material

Additional data file 1 Tested miRNAs and their expression levels and fold changes Click here for file

Additional data file 2 Data conditioning on PMI, pH, and hemisphere Click here for file

Additional data file 3 qRT-PCR results Click here for file

Additional data file 4 Characterization and microarray results on rats treated with haloperidol Click here for file

Additional data file 5 Host mRNA data Click here for file

Additional data file 6 Putative motifs within regions upstream of some distinguished pre-miRNAs Click here for file

Additional data file 7 Primer sequences Click here for file

## Figures and Tables

**Table 1:** Differentially expressed miRNAs from the prefrontal cortex of subjects with schizophrenia compared to psychiatrically healthy subjects

miRNA	Fold change	Chromosome location(s)
hsa-miR-26b	0.63	2q35
hsa-miR-30b	0.68	8q24.22
hsa-miR-29b	0.69	1q32.2, 7q32.3
hsa-miR-195	0.73	17p13.1
hsa-miR-92	0.76	13q31.3, Xq26.2
hsa-miR-30a-5p	0.79	6q13
hsa-miR-30d	0.80	8q24.22
hsa-miR-20b	0.81	Xq26.2
hsa-miR-29c	0.82	1q32.2
hsa-miR-29a	0.82	7q32.2
hsa-miR-212	0.82	17p13.3
hsa-miR-106b	1.77	7q22.1
hsa-miR-7	0.70	9q21.32, 15q26.1, 19p13.3
hsa-miR-24	0.79	9q22.32, 19p13.12
hsa-miR-30e	0.89	1p34.2
hsa-miR-9-3p	0.77	1q22, 5q14.3, 15q26.1

**Table 2:** KEGG pathways of gene categories that are over-represented by targets of two or more miRNAs distinguished by schizophrenia

KEGG Pathway Term	N	%	*P *value
HSA04810:REGULATION OF ACTIN CYTOSKELETON	43	2.81	1.7E-07
HSA04510:FOCAL ADHESION	45	2.94	3.5E-07
HSA04010:MAPK SIGNALING PATHWAY	41	2.68	3.9E-05
HSA04512:ECM-RECEPTOR INTERACTION	17	1.11	0.0029
HSA04070:PHOSPHATIDYLINOSITOL SIGNALING	18	1.17	0.0076
HSA04020:CALCIUM SIGNALING PATHWAY	28	1.83	0.0093
HSA00271:METHIONINE METABOLISM	6	0.39	0.0099
HSA04540:GAP JUNCTION	16	1.04	0.0109
HSA04530:TIGHT JUNCTION	18	1.17	0.0173
HSA04910:INSULIN SIGNALING PATHWAY	21	1.37	0.0193
HSA04630:JAK-STAT SIGNALING PATHWAY	22	1.44	0.0326
HSA04710:CIRCADIAN RHYTHM	5	0.33	0.0370

N, number of potential target genes in pathway; %, percent of pathway genes that are targeted by differentiated miRNAs.

**Table 3:** Demographics

Subject	Age (years)	PDx	Sex	PMI	pH	Hemisphere
1003	51-60	Ctrl	F	24	5.8	Right
1006	51-60	Ctrl	M	24.2	6.53	Left
1008	61-70	Ctrl	F	22.5	6.26	Left
1013	31-40	Ctrl	M	18.75	6.68	Right
1014	31-40	Ctrl	M	20	5.97	Left
1020	71-80	Ctrl	M	20.53	6.05	Right
1021	31-40	Ctrl	M	25.67	6.33	Right
1022	80+	Ctrl	M	7.42	6.39	Right
1024	71-80	Ctrl	M	20.92	6.74	Left
1025	71-80	Ctrl	F	23.91	6.67	Right
1026	31-40	Ctrl	M	28.83	6.53	Left
1028	61-70	Ctrl	F	24.25	6.4	Right
1029	61-70	Ctrl	F	7.42	6.03	Right
1030	41-50	Ctrl	M	18.33	6.78	Left
1032	41-50	Ctrl	M	24.13	6.01	Left
1033	80+	Ctrl	M	28.58	6.42	Right
1034	31-40	Ctrl	M	16.6	6.24	Left
1035	31-40	Ctrl	M	24.5	6.26	Left
1047	61-70	Ctrl	M	15.3	6.88	Right
1057	31-40	Ctrl	M	28	6.5	Right
1066	21-30	Ctrl	M	18.25	7.06	Left
1001	61-70	SZ	M	22.1	6.43	Right
1009	71-80	SZ	F	24	6.08	Right
1016	61-70	SZ	M	22.35	6.55	Right
1036	41-50	SZ	M	19	6.05	Left
1037	31-40	SZ	M	28	6.25	Left
1038	41-50	SZ	M	18.1	6.26	Left
1039	71-80	SA	F	13.4	6.81	Left
1040	41-50	SZ	M	18.5	6.31	Left
1042	21-30	SZ	M	16	6.75	Right
1043	41-50	SZ	M	27.1	6.64	Right
1044	41-50	SZ	M	19.25	6.57	Right
1052	80+	SZ	F	23.25	5.91	Right
1060	71-80	SZ	F	21.75	6.65	Right
1061	41-50	SA	F	33.78	6.63	Left
1065	41-50	SZ	M	19.08	6.6	Left

Ctrl, control; F, female; M, male; PDx, primary diagnosis; PMI, postmortem interval hours; SA, schizoaffective; SZ, schizophrenia.
