# Supplementary material for: microRNA expression in the prefrontal cortex of individuals with schizophrenia and schizoaffective disorder
Source: Genome Biol. 2007 Feb 27;8(2):R27. doi: 10.1186/gb-2007-8-2-r27 (PMC1852419; doi:10.1186/gb-2007-8-2-r27)
Supplement: Additional data file 7 — Primer sequences [file gb-2007-8-2-r27-S7.doc]

Forward miRNA primers:

|  | Primer |
| --- | --- |
| let-7g | UGAGGUAGUAGUUUGUACAGU |
| miR-128a | UCACAGUGAACCGGUCUCUUUU |
| miR-195 | UAGCAGCACAGAAAUAAUGGC |
| miR-24 | UGGCUCAGUUCAGCAGGAACAG |
| miR-26b | UUCAAGUAAUUCAGGAUAGGU |
| miR-29b | UAGCACCAUUUGAAAUCAGU |
| miR-30a-3p | CUUUCAGUCGGAUGUUUGCAGC |
| miR-30b | UGUAAACAUCCUACACUCAGC |
| miR-30e-5p | UGUAAACAUCCUUGACUGGA |
| miR-302a | UAAGUGCUUCCAUGUUUUGGUGA |
| miR-7 | UGGAAGACUAGUGAUUUUGUU |
| miR-92 | UAUUGCACUUGUCCCGGCCUGU |
| U6 FOR | CGCUUCGGCAGCACAUAUAC |
| U6 REV | UUCACGAAUUUGCGUGUCAU |
| Universal reverse primer | GCAGCACAGAATTAATACGACTCAC |
